# Dissociating two aspects of human 3D spatial perception by studying fighter pilots

**DOI:** 10.1038/s41598-023-37759-w

**Published:** 2023-07-12

**Authors:** Gily Ginosar, Ehud D. Karpas, Idan Weitzner, Nachum Ulanovsky

**Affiliations:** 1 Department of Brain Sciences, Weizmann Institute of Science, 76100 Rehovot, Israel; 2 Sackler School of Medicine, Tel Aviv University, 69978 Tel Aviv, Israel

**Keywords:** Cognitive neuroscience, Learning and memory

## Abstract

Human perception of 3D space has been investigated extensively, but there are conflicting reports regarding its distortions. A possible solution to these discrepancies is that 3D perception is in fact comprised of two different processes—perception of traveled space, and perception of surrounding space. Here we tested these two aspects on the same subjects, for the first time. To differentiate these two aspects and investigate whether they emerge from different processes, we asked whether these two aspects are affected differently by the individual's experience of 3D locomotion. Using an immersive high-grade flight-simulator with realistic virtual-reality, we compared these two aspects of 3D perception in fighter pilots—individuals highly experienced in 3D locomotion—and in control subjects. We found that the two aspects of 3D perception were affected differently by 3D locomotion experience: the perception of 3D traveled space was plastic and experience-dependent, differing dramatically between pilots and controls, while the perception of surrounding space was rigid and unaffected by experience. This dissociation suggests that these two aspects of 3D spatial perception emerge from two distinct processes.

## Introduction

**Figure 1:**
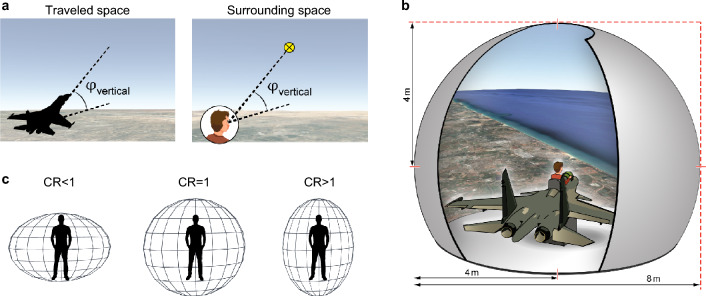
Experimental setup: highly-realistic immersive flight simulator. (**a**) Traveled space versus surrounding space. Left—Cartoon depicting traveled space: the angles and distances of motion, with respect to the world. Right—Cartoon depicting surrounding space: the angles and distances surrounding the subject, with respect to its body. (**b**) A cartoon depicting the experimental setup: a flight simulator of an F-15 fighter jet. The simulator consisted of a real airplane cockpit surrounded by a half-dome large screen. The screen was 8 m in diameter, creating a wide 210° horizontal view × 105° vertical view for the subject sitting inside the immobile cockpit. Subject and airplane were not drawn here to scale (additionally, the actual simulator contained only the front half of the airplane). (**c**) A cartoon illustrating the compression ratio (CR). Middle: CR = 1 corresponds to the vertical and horizontal dimensions being perceived identically (see circle). Left: CR < 1, a vertically-compressed perception (see ellipse) means that vertical angles are overestimated. Right: CR > 1, a horizontally-compressed perception (see ellipse) means that horizontal angles are overestimated.

The world is three-dimensional (3D), and both animals and humans need to perceive 3D space. Nonetheless, humans were shown to perceive 3D space in a distorted manner, compressing the vertical dimension^1–9^. However, although the perception of 3D space is commonly regarded and investigated as a single process, we suggest that it is actually comprised of at least two separate aspects: the perception of traveled space (Fig. 1a, left)—the distances and angles of self-motion through space, which are used for path integration; and the perception of the surrounding space (Fig. 1a, right)—the distances and angles to objects in one’s surrounding, relative to one’s self, which are used for scene analysis and self-triangulation. Interestingly, previous studies that investigated the perception of 3D surrounding space, unambiguously reported a distorted, anisotropic perception of 3D space^1–3,5,6,8,9^. By contrast, studies that utilized the perception of 3D traveled space varied in their reports, revealing a mixed picture of both isotropic and anisotropic perception^4,7,10,11^. These conflicting findings may indicate that the perception of 3D traveled space and the perception of 3D surrounding space result from separate and distinct processes—suggesting fundamental differences between these aspects of perception. These differences may have gone unnoticed because classically each study addressed only one of these two aspects of 3D perception. Here, we examined both of these two different aspects of the perception of 3D space in the same subjects, and set out to dissociate them. Inspired by how locomotion patterns shape spatial perception in the animal kingdom^12–17^, we investigated whether the two aspects of perception are differentially shaped by locomotion patterns.

To this end, we studied the two aspects of perception in both fighter pilots and control subjects, using a realistic F-15 fighter jet virtual-reality flight simulator (Fig. 1b). Fighter pilots are highly experienced in 3D locomotion and in navigation through 3D space. When flying a fighter jet, they experience more degrees of freedom in their 3D locomotion than when traveling horizontally on the ground. These observations suggest that if experience is able to shape the perception of traveled space, it would be apparent in these expert individuals. Likewise, fighter pilots are highly experienced in assessing and interacting with things surrounding them beyond the horizontal surface: pilots are relying on visual cues on the land beneath them to navigate, and also need to be constantly aware of the relational 3D position of other airplanes flying below, above and behind them—whether they are friends or foes. Here we tested the two aspects of 3D spatial perception in pilots versus control subjects, and found that the two aspects of perception indeed differ depending on the individual’s experience. The perception of traveled space exhibited plasticity and was strongly shaped by the individual’s experience in 3D navigation: while the control group exhibited vertically-compressed perception, the expert pilots did not exhibit any compression, but rather showed isotropic perception of space. By contrast, the 3D perception of the surrounding space was unaffected by individual experience: a vertically-compressed perception was seen in all subjects regardless of experience in 3D. Taken together, we show here a differential result: one aspect of 3D spatial perception was plastic and was strongly affected by experience, while another aspect was not. Our results therefore suggest that human perception of 3D space is composed of two distinct aspects—likely resulting at least in part from different mechanisms, since they differ in their sensitivity to the individual’s life-experience.

## Results

We used a highly realistic virtual reality flight-simulator to visually simulate 3D flight in an F-15 fighter jet. The flight-simulator consisted of a real, full-sized F-15 cockpit surrounded by a half-dome with a very large screen—8 m in diameter (Fig. 1b). Previous studies have shown that in order to create a realistic subjective feeling of motion in virtual reality, it is crucial to use wide-field images that cover the visual periphery^18^. Here, the subject sat inside an immobile cockpit, surrounded by a half-dome screen which created a very wide field of view: 210° horizontal view and 105° vertical view (60° above the horizon and 45° below it), without any vestibular stimuli. The display consisted of a realistic, highly-detailed world-view animation that was projected onto the screen (resembling Google Earth, but with much higher resolution). All subjects reported that the simulated flight created a highly immersive flight experience.

To test the effect of experience on perception, we studied two groups of participants. The test group was composed of fighter pilots and navigators (n = 16; age 38.5 ± 5.6 years, mean ± s.d.; 15 males, 1 female; see “Methods”), all with extensive experience in volumetric flight in 3D space, with > 1000 flight hours each. Of this group, 9 were fighter pilots (7 fighter-jet airplane pilots and 2 helicopter pilots); and 7 were fighter-jet airplane navigators. The control group were age-matched and gender-matched subjects with no prior flight experience (n = 16; age 38.1 ± 10.6 years, mean ± s.d.).

To assess whether perception of 3D space is isotropic and undistorted or anisotropic and compressed, we defined a compression ratio (CR)—to provide a metric for the relation between the vertical and horizontal axes when the subject perceived them as equal (“Methods”, Fig. 1c). We used the CR to assess the perception of both traveled space and surrounding space (“Methods”). CR = 1 indicates that the horizontal and vertical axes are perceived similarly (isotropically), with no perceptual distortions (Fig. 1c, middle). By contrast, CR < 1 indicates that the vertical angles are overestimated, such that the vertical axis is perceived as ‘compressed’, while CR > 1 indicates that the vertical axis is perceived as expanded (Fig. 1c, left and right).

### Perception of 3D traveled space is un-distorted in experienced pilots but distorted in control subjects

**Figure 2:**
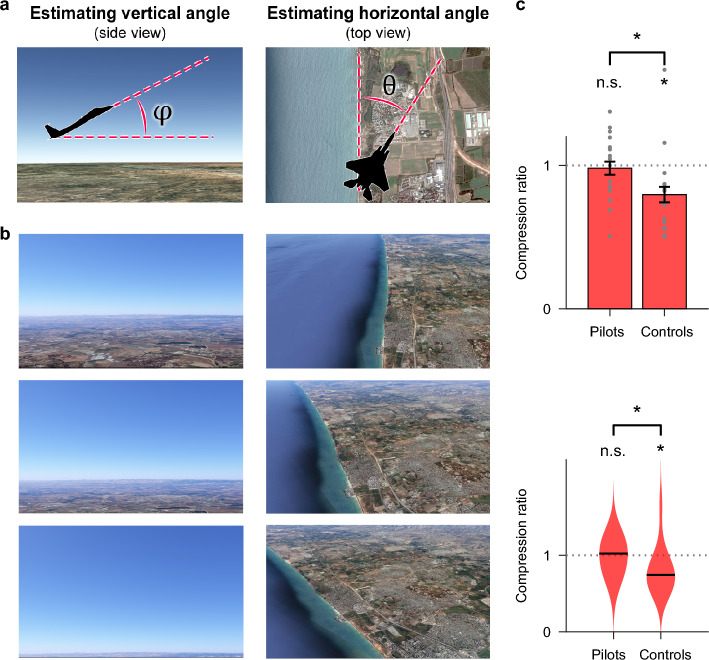
The perception of 3D traveled space was influenced by experience. Pilots exhibited un-distorted perception of 3D traveled space while control subjects exhibited compressed 3D perception. (**a**) A cartoon depicting the experimental design for studying perception of 3D self-motion through 3D traveled space (Experiment 1). Left: Subjects were flown at a vertical climb angle φ through 3D space and were asked to verbally assess the vertical angle (the vertical climb was visual-only and not vestibular, but was perceived by the subjects as a true climb). Right: subjects were flown at a horizontal angle θ relative to a clearly observed straight shoreline, and were asked to verbally assess the horizontal angle. The vertical and horizontal perceptions of 3D self-motion were assessed using a compression ratio (CR) (see “Methods”). **b** Pictures (from Google Earth) resembling the subjects’ view in different climb epochs and horizontal epochs: plotted for illustration purposes. We note that the actual field of view in the flight simulator was much broader than shown here—and consequently, the ground remained always in sight during climb epochs (as the view was broader in all directions than shown here on the left), and in addition, both the land and the sea (i.e. both sides of the shoreline) always remained in sight during horizontal epochs (right). (**c**) The CR of the pilots group and of the control group while assessing 3D self-motion in 3D space. Dotted horizontal gray line indicates CR = 1. Top panel – Error bars, mean ± s.e.m.; gray dots show individual subjects. Statistical tests: controls: ‘*’, mean CR = 0.796, t = − 2.79, P = 0.014 using a paired two-sided t-test, when compared to CR = 1; pilots: ‘n.s’, mean CR = 0.98, t = − 0.33, P = 0.75—non-significant difference when compared to CR = 1; comparison between groups: ‘*’, t = 1.95, P = 0.03 using a two-sample one-sided t-test (a one-sided test was used because it was clear from the data that the pilots’ CR is larger than the control subjects’ CR). Bottom panel—violin plots; horizontal black bars indicate median; controls: ‘*’, P_wilc_ = 0.013 using paired two-sided Wilcoxon signed-rank test, when compared to CR = 1; pilots: ‘n.s’, P_wilc_ = 0.76—non-significant difference when compared to CR = 1; comparison between groups: ‘*’, P_wilc_ = 0.014 using two-sample one-sided Wilcoxon rank-sum test.

We first asked whether human perception of self-travel through 3D space (Fig. 1a, left) is affected by experience of locomotion and navigation through 3D space. Specifically, we investigated whether experience changes the anisotropic manner in which humans perceive 3D space. To study perception per se, disentangled from action, we studied the subjects (both pilots and controls) as they were flown passively in the simulated 3D space. Each trial consisted of two epochs. First, a baseline epoch—a ‘straight and level’ virtual flight in which the airplane flew parallel to the ground at an altitude of 3–7 km. Next, a test epoch—in which the flight was conducted under two possible conditions: (i) various vertical climbing angles relative to the horizon (Fig. 2a,b, left); and (ii) various horizontal angles relative to a shoreline—a very salient environmental linear feature (Fig. 2a,b, right; see Supplementary Table S1 for the list of trials). In each flight epoch, the subjects were instructed to verbally estimate the vertical angle φ relative to the horizon (during climb epochs with actual values of φ = 30°/45°/60°: Fig. 2a, left—Cartoon; Fig. 2b, left—Examples illustrating the subject’s visual field in different vertical climbing angles; note that the field-of-view in the flight simulator was much broader than shown here, and hence the ground always remained in sight during climb epochs); or to estimate the horizontal angle θ relative to the shoreline (during horizontal epochs of θ = 30°/45°/60°: Fig. 2a, right—Cartoon; Fig. 2b, right—Examples illustrating the subject’s visual field in different horizontal angles relative to shoreline; note that both sides of the shoreline always remained in sight during the horizontal epochs). In order to overcome possible biases in verbal reports, we compared the reported angles in a pairwise manner (rather than treating each absolute report separately): that is, we did not focus on the values of the reported angles themselves, but rather compared the ratio between the reported angles φ and θ when the actual angles were identical. CR was defined as:

$$ {\text{CR }} = \, \left( {\varphi_{{{\text{real}}}} /\varphi_{{{\text{estimated}}}} } \right) \, / \, \left( {\theta_{{{\text{real}}}} /\theta_{{{\text{estimated}}}} } \right), $$

and was computed for each of the three angles used in the experiment (see “Methods”), and then averaged over trials to obtain the traveled-space CR for each subject. Control subjects displayed a traveled-space CR significantly smaller than CR = 1 (Fig. 2c, right bar: mean CR = 0.796; top panel—right bar graph: paired two-sided t-test versus CR = 1, t = − 2.79, P = 0.014; bottom panel—right violin graph: paired two-sided Wilcoxon signed-rank test, P_wilc_ = 0.013; we note that given the small sample size [n = 16 in each group] we report both mean and median statistics). This indicates a distorted anisotropic perception of self-motion through 3D space, with overestimation of vertical angles. Surprisingly, experienced pilots exhibited a CR that was not significantly different from CR = 1 (Fig. 2c, left bar: mean CR = 0.98; top panel—left bar graph: paired two-sided t-test versus CR = 1, t = − 0.33, P = 0.75; bottom panel—left violin graph: paired two-sided Wilcoxon signed-rank test, P_wilc_ = 0.76)—and the CR of pilots was significantly larger than the CR for the control group (two-sample one-sided t-test, t = 1.95, P = 0.03; one-sided Wilcoxon rank-sum test, P_wilc_ = 0.014; see Supplementary Fig. S1 for the reported angles for all the subjects from both groups). Results did not differ significantly between pilots (n = 9) and airplane navigators (n = 7; see Supplementary Fig. S2)—hence we pooled these groups together, and refer to them below as ‘pilots’. Further, prior familiarity with the setup (within the pilots/navigators group) did not affect the results (Supplementary Fig. S2). The undistorted isotropic perception of 3D traveled space in pilots that are highly-experienced in 3D navigation, but not in control subjects, indicates the existence of an aspect of human perception of 3D space that is altered by an individual’s 3D experience, namely—the perception of self-motion through 3D space.

### Perception of the surrounding 3D space is vertically-compressed, in both pilots and control subjects

**Figure 3:**
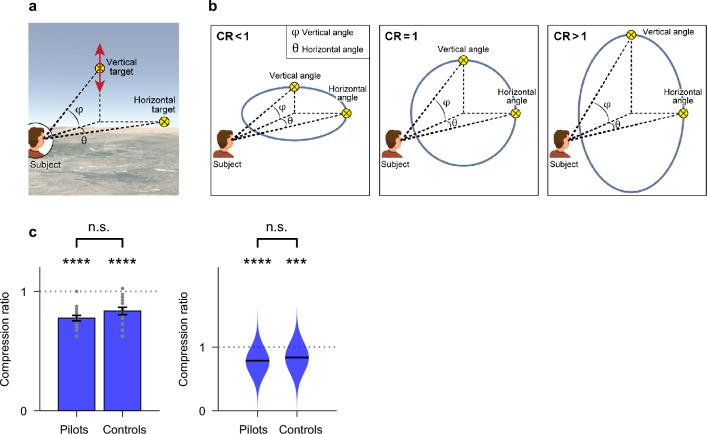
The perception of 3D surrounding space was unaffected by experience. (**a**) A cartoon depicting the experimental design for testing the perception of 3D surrounding space (Experiment 2). The subject sat facing the screen. Two targets appeared on the screen: a horizontal target at a horizontal shift θ from a straight-ahead point, and a vertical target at a vertical shift φ from a straight-ahead point. The subject was asked to look around and identify the targets. During the experiment, the subject was asked to verbally instruct the experimenters to move the vertical target up or down, while the horizontal target remained stationary—this continued until the subjects reported that they perceived the vertical shift of the vertical target (φ_final_) to be identical to the horizontal shift of the horizontal target (θ). We quantified vertical versus horizontal perception with a compression ratio (CR), as follows: CR = φ_final_/θ (see “Methods” and Supplementary Fig. S3). (**b**) A cartoon illustrating the compression ratio (CR). Middle: CR = 1 corresponds to vertical angles and horizontal angles perceived identically. Left: CR < 1, a vertically-compressed perception means that vertical angles are overestimated. Right: CR > 1, a horizontally-compressed perception means that horizontal angles are overestimated. (**c**) The measured CR of the pilots group and of the control group. Dotted horizontal gray line indicates CR = 1. Left panel—Error bars, mean ± s.e.m.; gray dots show individual subjects. Statistical tests: paired two-sided t-test versus CR = 1: Pilots: ‘****’, mean CR = 0.78, t = − 9.82, P = 6 × 10^–8^; Controls: ‘****’, mean CR = 0.84, t = − 5.32, P = 9 × 10^–5^; ‘n.s’, non-significant difference between the groups using a two-sample two-sided t-test: t = − 1.55, P = 0.13. Right panel—violin plots; paired two-sided Wilcoxon signed-rank test versus CR = 1; black horizontal lines indicate median. Pilots: ‘****’, P_wilc_ = 6 × 10^–5^; Controls: ‘***’, P_wilc_ = 8 × 10^–4^; ‘n.s’, non-significant difference between the groups using a two-sample two-sided Wilcoxon rank-sum test: P_wilc_ = 0.15.

Next, we asked whether human perception of one’s surrounding 3D space (Fig. 1a, right) is affected by experience of locomotion and navigation through 3D space. To this end, we performed a different experiment than before—on the same set of subjects. Participants were seated inside the cockpit, with two targets appearing on the screen surrounding them—a vertical target shifted upwards above eye-level, and a horizontal target that was horizontally shifted to the side of the subject (Fig. 3a, and Supplementary Fig. S3). In each trial, the horizontal target was placed at θ = 40°, and the vertical angle φ_initial_ was chosen at random with a value that greatly differed from θ (see “Methods” and Supplementary Table S2 for the list of trials). Both targets were visible and subjects were instructed to move their heads and look around them as needed in order to look at the targets directly. Then, the participants were asked to verbally instruct the experimenters by how much to change the vertical target’s position (by a “little/medium/large amount”), and could continue to fine-tune the vertical target’s position until the subjects declared that they perceive the vertical shift as identical to the horizontal shift (Fig. 3a). Subjects did not receive any feedback on their choices. The final vertical shift that the subject declared as identical to the horizontal shift was termed φ_final_ (see Supplementary Fig. S3, step 5). The compression ratio (CR) was defined as:

$$ {\text{CR }} = \, \varphi_{{{\text{final}}}} / \, \theta $$

CR = 1 corresponds to isotropic perception (Fig. 3b, middle); CR < 1 indicates that the vertical axis is perceived as overestimated, or ‘compressed’, while CR > 1 indicates that the vertical axis is perceived as expanded (Fig. 3b, left and right). Both pilots and control subjects displayed a CR significantly lower than 1 (Fig. 3c; Pilots—left panel: mean CR = 0.78, paired two-sided t-test versus CR = 1, t = − 9.82, P = 6 × 10^–8^; Pilots—right panel: paired two-sided Wilcoxon signed-rank test versus CR = 1, P_wilc_ = 6 × 10^–5^; Controls—left panel: mean CR = 0.84, paired two-sided t-test versus CR = 1, t = − 5.32, P = 9 × 10^–5^; Controls—right panel: paired two-sided Wilcoxon signed-rank test versus CR = 1, P_wilc_ = 8 × 10^–4^). Both groups displayed similar levels of 3D spatial distortion (Fig. 3c; Left, two-sided two-sample t-test, t = − 1.55, P = 0.13; Right, Wilcoxon rank-sum test, P_wilc_ = 0.15). Results did not differ significantly between pilots (n = 9) and airplane navigators (n = 7; see Supplementary Fig. S2)—hence we pooled these groups together. Further, prior familiarity with the setup (within the pilots/navigators group) did not affect the results (Supplementary Fig. S2). The compression results were quite robust to variations in test conditions: (i) Varying which target (vertical or horizontal) is being adjusted: The standard testing conditions comprised of a fixed unmovable horizontal target at θ = 40° and an adjustable vertical target that was moved until it was perceived to be matching the horizontal one. In this control, the subjects moved the horizontal target while the vertical target was fixed at vertical φ = 40° (Supplementary Fig. S4a). The CR attained under the control condition did not differ significantly from the CR in the main experiment (paired two-sided t-test: t = 0.52, P = 0.6; paired two-sided Wilcoxon sign rank test: P_wilc_ = 0.39; n = 32 for all subjects). (ii)–(iii) Control for the side at which the horizontal target was presented: (ii) Handedness of subject relative to the fixed target: The fixed target was placed on the side of the subject’s dominant hand versus the side of the non-dominant hand (Supplementary Fig. S4b). The CR attained under the control condition did not differ significantly from the CR in the main experiment (paired two-sided t-test: t = − 0.32, P = 0.74; paired two-sided Wilcoxon sign rank test: P_wilc_ = 0.37; n = 32 for all subjects). (iii) Side of fixed target: The fixed target was placed to the left of the subjects, at θ = − 40°, as opposed to the standard positioning to the right of the subjects, at θ =  + 40° (Supplementary Fig. S4c). Here the CR with right target was slightly smaller than the CR with left target (paired two-sided t-test: t = − 2.03, P = 0.0507; paired two-sided Wilcoxon sign rank test: P_wilc_ = 0.023; n = 32 for all subjects). Although this difference was significant (in the Wilcoxon test, but not in the t-test), it was a very small difference—a reduction of only 5.4%. While this reduction could reflect side biases, it did not appear in the handedness control, and hence this possibility seems less likely. We note that due to time constraints on the usage of the flight simulator, each of these control variables was tested once per each subject (see Supplementary Table S2). Taken together, these results suggest that the biased, anisotropic perception of surrounding 3D space is a very robust and rigid human characteristic, which is un-affected by an individual’s experience in 3D perception and locomotion.

### The perceptual distortion of surrounding 3D space is egocentric and not allocentric

We next investigated whether the compression effect, which distorts the perception of surrounding 3D space, occurs in an egocentric reference frame (relating to the subject’s body), or whether it is an allocentric effect (relating to the outside world and the horizon). These reference frames are difficult to decouple, because many animals, from flies to motorcycle drivers, make an effort to keep their eyes aligned with the horizon, i.e. display near-zero head-roll, regardless of body orientation^15,19^—and in so doing, they align the egocentric frame of reference (coupled to the body) and allocentric frame of reference (coupled to the outside world: the horizon). Consequently, an egocentric and an allocentric compression effects would appear identical. Our unique set-up enabled us to decouple these two reference-frames by simulating an airplane’s roll maneuver, thus mis-aligning the horizon and the subject’s body—and then testing which of these reference frames explains best the compression effect.

**Figure 4:**
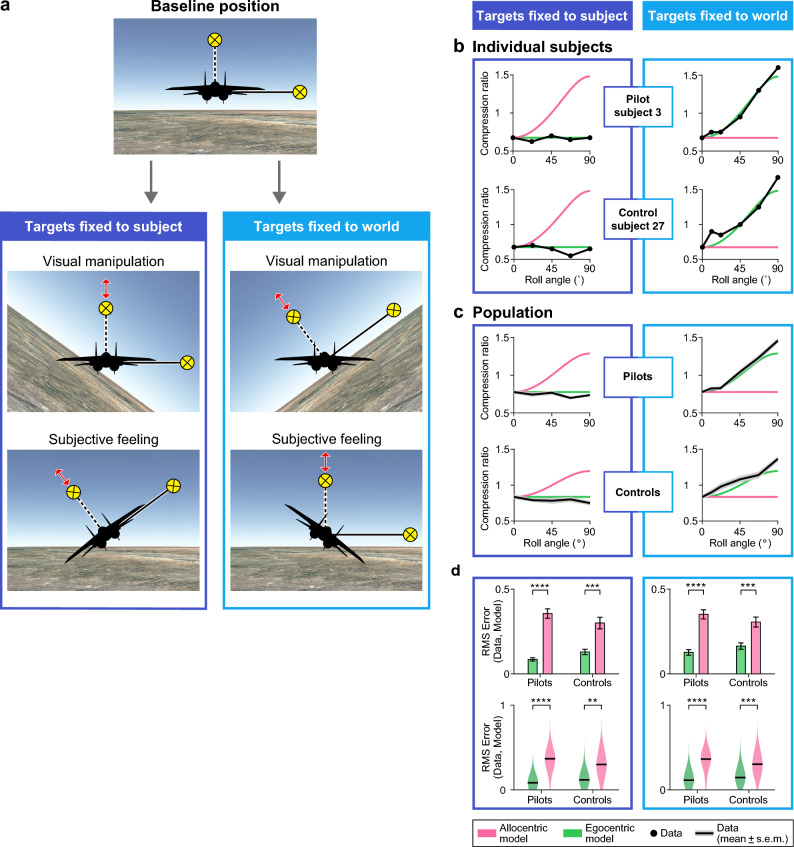
Anisotropic perception of 3D space is egocentric and not allocentric, in both pilots and controls. (**a**) A cartoon describing the experiment that tested egocentric versus allocentric compression (a variation on Experiment 2: see “Methods”). Top—the baseline experimental design, as depicted in Fig. 3a. Below—four panels that depict two different roll manipulations (columns) performed to distinguish between egocentric and allocentric compressions. Left column (dark blue): subject rolling, with targets rolling together with the subject. Right column (light blue): Subject rolling, while targets remained fixed in space with respect to the world (i.e. with respect to the horizon). Middle row—the immersive visual manipulation performed on the subjects sitting inside the stationary cockpit. Bottom row—the subjective feeling of a roll maneuver that the visual manipulation elicited in the subjects. Red arrows indicate the direction in which the subjects could move the target. (**b**) Compression ratios (CR) measured at different roll angles: representative examples from two individual subjects. Left column: targets fixed to the subject; Right column: targets fixed to the world. Top row: example of a pilot subject; Bottom row: example of a control subject. Black dots are the subject’s CR values at different roll angles. Magenta line: expected CRs for each scenario using a model of allocentric CR (compression coupled to the horizon, see “Methods”). Green line: expected compression ratios for each scenario using a model of egocentric CR (compression coupled to the subject). (**c**) Average population data, plotted as in panel (**b**). Shown are average compression ratios at different roll angles, pooled across each of the two populations (top—pilots, bottom—controls), when the targets were rolling together with the subjects (left column) versus when the targets remained fixed to the world (right column). Black line and gray area, mean ± s.e.m. of the data; magenta line, prediction of allocentric model; green line, prediction of egocentric model. (**d**) Population statistical analyses: comparing the data to the egocentric and allocentric models. Shown is a comparison of distances (RMSE—root mean square error) between the CRs of subjects (Data) and CRs expected from the same roll angles under the egocentric model (Model, green) versus under the allocentric model (Model, pink). Left column: subject rolling, with targets rolling together with the subject. Right column: Subject rolling, while targets remained fixed in space with respect to the world (fixed to the horizon). Top row: Error bars, mean ± s.e.m.; here we compared the means using a paired two-sided t-test; left: pilots: ‘****’, t = − 9.46, P = 1 × 10^–7^; controls: ‘***’, t = − 4.67, P = 3 × 10^–4^; right: pilots: ‘****’, t = − 8.2, P = 6.7 × 10^–7^; controls: ‘***’, t = − 4.9, P = 2 × 10^–4^. Bottom row: Violin plots: same data as in top row, but plotted as a violin plot; here we compared the medians using a paired two-sided Wilcoxon signed-rank test; left: pilots: ‘****’, P_wilc_ = 6 × 10^–5^; controls: ‘**’, P_wilc_ = 1.3 × 10^–3^; right: pilots: ‘****’, P_wilc_ = 6 × 10^–5^; controls: ‘***’, P_wilc_ = 8 × 10^–4^.

Subjects’ body orientation was experimentally dissociated from the orientation of the horizon by simulating a roll maneuver at different roll angles. While the subject felt the airplane is rolled with respect to the ground, we conducted the ‘surrounding space’ compression experiment (Fig. 4a, bottom row). We conducted two ‘roll scenarios’, in which the visual display of the horizon was tilted (in roll angles varying from 11° to 90° roll; 9 roll trials per subject, see “Methods” and Supplementary Table S2), while the subjects were physically stationary. Although this manipulation was purely visual, the highly-immersive wide-field virtual reality system created a very strong illusion, making the subject feel as if it was the subject itself that had rolled with the aircraft relative to the horizon. In one roll-scenario the targets either rolled with the subject (Fig. 4a–d, left column); in a second roll-scenario the targets were fixed to the world reference-frame (Fig. 4a–d, right column). In both roll-scenarios, the subjects were instructed to keep their heads upright and refrain from tilting their heads with the tilted visual horizon. The subjects’ task was to adjust the vertical target with respect to the horizontal target—as in the previous experiment (Fig. 3). For each of these tests, we subsequently modeled what would be the compression ratio of the displayed targets in different roll angles under two possible models: (i) a model of an egocentric compression effect, rolling with the subjects, and (ii) a model of allocentric compression effect, fixed to the horizon (see “Methods”, and Supplementary Fig. S5). Under both roll-scenarios, the CRs for both pilots and control subjects displayed excellent agreement with the egocentric model: whenever the targets were fixed (yoked) to the subject, the compression in perception (the CR value) remained stable, regardless of the magnitude of the roll angle (Fig. 4b–d, left). Further, whenever the targets were fixed to the world (fixed to the horizon), the compression effect closely followed the prediction of the egocentric model—but not the allocentric model (Fig. 4b, right—examples; Fig. 4c, right—population: note the data [black] align well with the egocentric model predictions [green] but not with the allocentric model predictions [magenta]). The fit of the data to the egocentric model was highly significantly better than the fit to the allocentric model, in both roll scenarios (Fig. 4d—statistics for the population: the root mean square (RMS) error between data and model is smaller [indicating better fit] for egocentric model predictions [green] than for allocentric model predictions [magenta]; Fig. 4d, left, targets fixed to subject—Top: paired two-sided t-test comparing the two models, pilots: t = − 9.46, P = 1 × 10^–7^; controls: t = − 4.67, P = 3 × 10^–4^; Bottom: paired two-sided Wilcoxon signed-rank test comparing the two models, pilots: P_wilc_ = 6 × 10^–5^; controls: P_wilc_ = 1.3 × 10^–3^; Fig. 4d, right; targets fixed to world—Top: paired two-sided t-test, pilots: t = − 8.2, P = 6.7 × 10^–7^; controls: t = − 4.9, P = 2 × 10^–4^; Bottom: paired two-sided Wilcoxon signed-rank test, pilots: P_wilc_ = 6 × 10^–5^; controls: P_wilc_ = 8 × 10^–4^). In particular, when the subject was rolled at 45° from the targets, the compression was gone, i.e. CR became equal to 1 (see Fig. 4b,c, right, note the data equal to 1 at roll angle = 45°; and Supplementary Fig. S5, bottom right, green ellipse). The compression then flipped to a horizontal compression at a roll of 90°, since it egocentrically followed the subject’s body, regardless of the horizon. These results demonstrate that the bias of vertical compression observed in humans is an egocentric effect—affixed to the subject’s head and body.

### 3D flight experience affects spatial perception of traveled space but not of surrounding space

**Figure 5:**
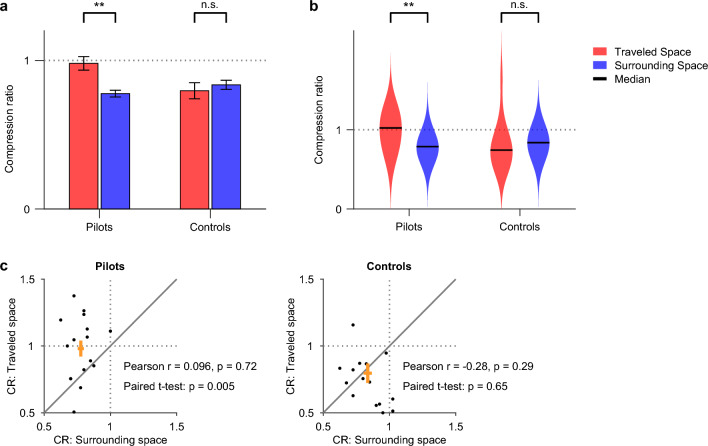
3D flight experience affects spatial perception of 3D traveled space but not of 3D surrounding space. (**a**,**b**) Compression ratios of pilots (**a**,**b**, left) and control subjects (**a**,**b**, right), comparing the perception of self-motion through 3D traveled space (red) versus the perception of 3D surrounding space (blue). Note that the raw data for individual subjects are shown in panel (**c**). (**a**) Error bars, mean ± s.e.m.: pilots: ‘**’, t = − 3.29, P = 5 × 10^–3^, using a paired two-sided t-test; controls: ‘n.s.’, t = 0.46, P = 0.65, non-significant difference. Dotted gray line indicates CR = 1. (**b**) Violin plots: same data as panel (**a**), but plotted as a violin plot. Horizontal black line indicates the median; pilots: ‘**’, P_wilc_ = 7 × 10^–3^, using Wilcoxon signed-rank test (paired); controls: ‘n.s.’, P_wilc_ = 0.50, non-significant difference. Dotted gray line indicates CR = 1. (**c**) Individual subjects’ data. Scatter plots show the traveled-space CR versus surrounding-space CR for each subject (dots), separately for pilots (left) and controls (right). Paired t-tests are indicated—these are the same t-tests as in panel (**a**), and they denote whether the data are significantly above the diagonal line (identity line). The Pearson correlation coefficients, r, are indicated, along with their P-values: there was no significant correlation between the surrounding-space CR and the traveled-space CR, neither for the pilots nor for the controls. One outlier control subject was not plotted here (but was included in the correlations and t-tests). Dotted gray lines indicate surrounding-space CR = 1 and traveled-space CR = 1. Orange crosses indicate the mean ± s.e.m. for each population in each of the axes.

Taking together the results of all the experiments, we found the following. In control subjects, spatial perception of both traveled and surrounding space was distorted, with the compression effect consistently over-estimating vertical shifts (Fig. 5a, right; paired two-sided t-test, t = 0.46, P = 0.65; Fig. 5b, right; paired two-sided Wilcoxon signed-rank test, P_wilc_ = 0.50). By contrast, pilots exhibited a distorted perception of surrounding space but not of traveled space (Fig. 5a, left; paired two-sided t-test, t = − 3.29, P = 5 × 10^–3^; Fig. 5b, left: paired two-sided Wilcoxon signed-rank test, P_wilc_ = 7 × 10^–3^; see Fig. 5c for per-subject comparison of traveled-space compression versus surrounding-space compression). These observations demonstrate that different aspects of human spatial perception are influenced differently by the individual’s 3D experience: The perception of self-motion through 3D space is sensitive to the individual’s experience, whereas the perception of 3D surrounding space seems to be general in humans and unaltered by experience.

## Discussion

Here we used an immersive highly-realistic flight simulator setup, to test expert subjects that are extensively trained in 3D flight and navigation. This unique combination enabled us to disentangle the two aspects of human perception of 3D space, by studying how experience in 3D perception, locomotion and navigation differentially affect these aspects.

In humans, studies of perception of 3D space that utilize the perception of surrounding space have demonstrated that humans perceive 3D space in a compressed anisotropic manner^1–3,5,6^. Other studies that utilized the perception of traveled space led to mixed results^4,7,10,11^. In animal work, it is widely thought that the spatial dimensionality in which a species navigates largely determines the species’ spatial perception^12–17^. Animals move and navigate through 3D space in different ways—e.g., crawling or walking or swimming or flying; those animals that are constrained to planar movement are thought to possess a biased anisotropic perception of 3D space, suiting their needs^13,16,20,21^. Conversely, the 3D perception of an animal that moves in a more isotropic manner will tend to be more isotropic, i.e. similar in the horizontal and vertical directions^12,14,16,17^. Inspired by this, we utilized a circumstance unique to humans—whereby this species contains a specialized sub-population of experts in 3D locomotion—in order to investigate whether experience of 3D motion and navigation in humans could dissociate between two aspects of the perception of 3D space: the perception of traveled space and the perception of surrounding space. We note that all subjects performed the ‘traveled space’ experiment first and the ‘surrounding space’ experiment second, and hence we cannot rule out that this ordering has influenced the results.

The perception of 3D angles of traveled space during self-motion through 3D volumetric space (Fig. 2)—which provide a crucial component of path integration—were plastic and were strongly influenced by experience. Our analysis revealed that in non-pilot controls, there is a compression-effect: control subjects exhibited a distorted, vertically-compressed perception of their own 3D locomotion. In striking contrast, pilots with extensive 3D experience did not show this compression effect when perceiving their own 3D locomotion.

By contrast, the perception of 3D surrounding space (Fig. 3) was unaltered by 3D flight experience, and exhibited compressed perception, which might be an innate human characteristic. Such anisotropic perception of surrounding angles has been previously demonstrated in humans tested in structured and compartmentalized spaces such as multi-floor buildings^1,2,6^. However, it has been suggested that this anisotropy might stem from the anisotropic setup of a building^7^. Here we showed that a compression effect is also present in an open-space setting, suggesting that human 3D spatial perception is vertically-compressed, regardless of the experimental setup. The compression was egocentric, yoked to the subjects’ body rather than to the outside visual world (Fig. 4).

The differences between pilots and controls (Fig. 5) may indicate not necessarily a difference in perception or perceptual learning, but rather a difference in other aspects of learning. For example, it may be that the pilots learned with experience how to correct for distortions of 3D space—namely, they might perceive space similarly to the controls, but they learned to correct for it differently than the controls. In other words, this learning may in fact be the learning of a ‘trick’ rather than perceptual learning. However, even if this is the case, it does not affect our central striking result—namely, that the ability of pilots to correct or learn 3D space differed between the surrounding space and the traveled space. This difference suggests that even if these results do not reflect perception per se, they do indicate the existence of two separate aspects of either the perception of 3D space or the representation of 3D space.

The different patterns of results in pilots versus controls (Fig. 5) could not be explained by a difference in familiarity with the flight simulator. First, the same results were seen in both pilots that had prior experience with the simulator, and in pilots that did not (Supplementary Fig. S2). Second and more importantly, pilots that did have experience with the flight simulator, were well-trained in the simulator in both aspects of 3D perception: in the traveled-space aspect (flight in the simulator), and in the surrounding-space aspect (interacting with targets in the simulator at all directions). Therefore, if the specific context of familiarity with the simulator had played a major role, we would expect the pilots to exhibit similar results in both traveled space and surrounding space experiments. However, all pilots showed a different result in traveled space versus surrounding space—specifically, they showed plasticity and learning only in the perception of traveled space, but not in the perception of surrounding space. This suggests that our results are not context-specific (setup-specific) but are likely domain-general.

Another potential critique is that the different patterns of results in pilots versus controls (Fig. 5) could be due not only to differences in experience of 3D spatial perception and locomotion—but also to differences in aptitude for 3D spatial perception. In other words, these differences could potentially be innate. Under this possibility, the selection process of air-force pilots is selecting individuals with high aptitude for 3D spatial perception. However, even if the difference between pilots and controls is innate, and is not experience-dependent as we proposed here, this does not alter the conclusion of our study—because if the perception of surrounding space and traveled space was due to a single process, then we would expect the pilots to exhibit similar results in both traveled space and surrounding space experiments. The finding of differential effects on traveled space versus surrounding space in pilots’ 3D spatial perception argues against this possibility—and points to two separate processes of 3D spatial perception. In other words, regardless of the experience-dependent versus innate explanation of the observed differences—our results support the same conclusion: namely, the existence of two distinct processes of human 3D spatial perception.

We speculate that these two distinct processes originate from two different brain regions. Specifically, we hypothesize that the 3D perception of traveled space—a task that requires information regarding one’s self position and movement through space—is created in the hippocampal formation, via place cells and grid cells, which are neurons that are activated as the animal travels through space^22–24^, and that were shown to represent 3D traveled space^23,25–28^. Importantly, the hippocampal formation is known for its plasticity—consistent with the plasticity that we observed in traveled-space experiments. We further hypothesize that the 3D perception of the surrounding space—a task that is essentially a visual assessment task—is created in the visual cortex, which is well known for analyzing visual scenes^29,30^. If this is true, then our results imply that the hippocampus is more plastic than the visual cortex. Future work would need to test the differences in long term experience-dependent plasticity in the hippocampus versus visual cortex. In principle, tackling these differences could be done using fMRI. However, two key issues must be addressed: First, the screen that is usually available in fMRI facilities is relatively small. By contrast, the effects reported here require an immersive experience with a wide-view screen. Second, fMRI is done while the patient is lying down, which alters the normal direction of gravity, and may therefore alter the reported effects, since the distinction between ‘horizontal’ and ‘vertical’ is altered when lying horizontally. Our results thus provide a first step for future mechanistic work aimed at elucidating in detail the nature of these two difference processes.

Taken together, through the use of a unique highly-realistic experimental setup, and a unique group of subjects who are exceptional 3D experts—fighter pilots—our experiments have explored human perception of 3D space, and have revealed aspects of human 3D spatial perception that are shaped differentially by experience. This study, which was performed in humans, may shed light on 3D spatial perception of other species as well—in which these questions may be more difficult to answer. We hypothesize that in other species, as in humans, 3D spatial perception is not a unitary phenomenon but consists of multiple processes—which are affected differently by experience.

## Methods

### Subjects

The subjects were 16 civilian pilots (15 males and 1 female; age 38.5 ± 5.6 years, mean ± s.d., age range 32–48; 12 were right-handed, 3 left handed, 1 ambidextrous). The control group comprised of 16 age-matched and gender-matched civilian technicians with no prior flight experience and no prior simulated-flight experience in any simulator (15 males and 1 female; age 38.1 ± 10.6 years, mean ± s.d., age range 27–57; 15 were right-handed, 1 left-handed). The number of subjects was dictated by the number of pilots who volunteered for the experiment. All subjects were naïve to the task. We removed from the analysis two additional civilian technicians who had extensive experience in this flight-simulator (i.e. initially we tested 18 technicians). All pilots had an experience of > 1200 flight hours each (range 1200–3000 flight hours in the air), and hundreds of hours of simulated flight in a 3D flight-simulator. Of this group, 9 were fighter pilots (7 jet airplane pilots and 2 helicopter pilots); and 7 were fighter-jet airplane navigators. All participants had good vision or good corrected vision. All subjects volunteered to the study and gave their written informed consent after oral and written instructions. They confirmed that they were free from any known neurological or physical illness. Participants did not receive any monetary reward for participation or for their performance. The experiment was conducted under the approval of the Institutional Review Board of the Weizmann Institute of Science (IRB no. 670-1), in accordance with the Helsinki Declaration. All pilots and technicians were civilians and volunteered as civilians.

### Set-up

The flight simulator was situated in a 9.5 × 10.5 m room. The simulator consisted of a cockpit of an F-15 fighter jet, placed in the middle of a large half-dome screen, with a diameter of 8 m. The subjects were seated in the front seat of the cockpit, on an elevated seat, which provided an unobstructed view of the visual display. The half-dome screen created a very wide field of view: 210° horizontal view and 105° vertical view (60° above the horizon and 45° below it). The setup provided a simulated highly-immersive realistic visual environment, created by a detailed world-view animation that was projected onto the screen (similar to Google Earth, but with much higher resolution), with no vestibular information. The subjects were instructed to naturally move their heads and gaze wherever they wished; the subjects’ eye and head movement were not tracked. Communication between the subject and the experimenters took place through a set of standard earphones and microphone. To gain familiarity with the setup prior to the experiment, all subjects initially experienced passive flight in the simulator that lasted ~ 10 min. Subjects spent a total of 60–75 min inside the cockpit during all the experiments and the familiarization-flight.

### Experimental design—Experiment 1: Assessing angles of traveled space

All subjects—both pilots and controls—were flown passively, in order to (i) circumvent the differences in flight abilities, and (ii) disentangle perception from action. Authors E.D.K., a trained fighter-jet navigator, or I.W., a trained fighter-jet pilot, sat at the back seat of this dual-seat cockpit and piloted the flight simulator, to enable the subjects to experience passive flight epochs while they were sitting in the front seat. The jet was initiated along Israel’s Mediterranean shoreline on each trial, and was then flown in a straight-and-level horizontal flight epoch for 5–20 s, followed by a randomly chosen trial. Individual trials lasted 0.5–1 min per trial; a total of 6 trials per subject (Supplementary Table S1). The trials consisted of either (i) a vertical climb at a fixed angle (vertical angles of 30°, 45° or 60°: Fig. 2a, left), or (ii) a straight-and-level horizontal flight at a particular azimuth relative to the well-defined and recognizable sea shoreline (horizontal angles of 30°, 45° or 60°: Fig. 2a, right). Each trial ((i) or (ii)) was followed by a straight-and-level epoch, to avoid sequential effects from previous trials; before each trial, subjects were notified when the airplane was at exactly straight-and-level flight parallel to the shoreline, to provide a reference direction. In each trial ((i) or (ii)), subjects were asked to declare the numerical value of the vertical angle at which the jet was flying relative to the horizon (at vertical trials—(i)), or at which horizontal angle the jet was flying relative to the shoreline (at horizontal trials—(ii)). We created a cognitive load on the subjects throughout this experiment, by using another task simultaneously: Multiple airplanes were appearing and disappearing in the field of view, and the participants were asked to identify the appearing airplanes and to verbally state their positions; this simultaneous task was aimed to prevent the subjects from developing complex alternative strategies for calculating their angle of flight—instead, it forced the subjects to declare the flight angle that they directly perceived. No feedback was given to the subjects regarding their performance.

### Experimental design—Experiment 2: Assessing angles of surrounding space

Subjects were seated in the flight-simulator’s cockpit. They were presented with a static view of the horizon at daylight, with no clouds, viewed from a 5-km altitude above ground. After subjects confirmed that they were sitting comfortably and that the screen was not obstructed, the experiment began. Individual trials lasted 2–4 min per trial; with a total of 14 trials per subject. The structure of each trial was as follows (see schematic in Supplementary Fig. S3): (1) A reference point (white dot) was displayed on the screen, in front of the subject, at the same simulated height as the subject—i.e. 5-km above ground—which corresponds to a 2° angular shift above the horizon. The subjects were then asked to adjust their seat height to allow the reference point to be displayed at their eye-level. (2) The reference point disappeared. (3) After a delay of at least 5 s, 2 targets (white dots) appeared: a horizontal target and a vertical target. The horizontal target appeared at the same height as the reference point (i.e. at eye-level) with a 40° shift to the right of the subject (except in control trials described below). The vertical target appeared simultaneously with the horizontal target, at the same azimuth where the reference point was (i.e. straight ahead of the subject), but at a random vertical angle (range of angles: 16°–52° above the horizon). (4) The horizontal target was held fixed, and subjects were asked to verbally instruct the experimenter how to move the vertical target up/down so that the vertical shift was identical to the horizontal (fixed) shift. Possible commands that the subjects could issue were related to the direction of motion of the vertical target (‘move up/down’) and the amount of movement (‘move by little/medium/large amount’). Little movement meant moving the target by 1°, while medium and large movements corresponded to moving 30% and 70% of the distance from the last location, respectively – thus creating a converging series of shifts. We allowed subjects to move the target as many times as they wanted, until: (5) They declared that they perceive the vertical and horizontal shifts of the two targets as being equal. As in Experiment 1, no feedback was given to the subjects regarding their performance. Three such trials were conducted per subject, and the CR values were averaged over the three trials. Trials were pseudo-randomly alternated with control trials and with roll trials (see below, and Supplementary Table S2).

Control experiments and analyses: To control for laterality effects, we employed three controls—all of which were performed by all subjects: First, we compared the standard trials, in which the horizontal target was on the right side of the subject, to a different test trial done for each of the subjects in which the horizontal target was placed on the left side of the subject (Supplementary Fig. S4c). Second, we compared trials in which the horizontal target was placed at the side of the subject’s dominant hand versus non-dominant hand (Supplementary Fig. S4b). Third, we compared the standard situation in which the horizontal target was fixed and the vertical target was moved, to the opposite situation in which the vertical target was fixed and the horizontal target was moved (Supplementary Fig. S4a). Each of these control tests was done for one trial in each of the subjects. No differences or only small differences in results were found between the standard setting and each of these 3 control settings (Supplementary Fig. S4). To control for familiarity with the setup, we compared those subjects within the pilots group that had prior experience with the specific flight simulator used here, to the pilots who did not have such experience: No differences in results were found due to familiarity with the setup (Supplementary Fig. S2).

Experiments to assess egocentric versus allocentric compression effect: This experiment was done as above, except that we simulated here a roll maneuver in various angles (22°, 45°, 67° and 90° roll; when targets were fixed to the world we used also 11° roll; a total of 9 trials per subject, see Supplementary Table S2)—such that the subjects felt as though the jet airplane in which they were sitting has rolled with respect to the ground. This subjective feeling was achieved by rolling the visual display of the outside world; the subjects were asked not to roll their head but keep it vertical relative to gravity. In some trials the targets rotated along with the subject’s egocentric reference frame, such that the targets were directly vertical or horizontal relative to the subject (see Fig. 4a, left; and Supplementary Fig. S5). In other trials the targets did not rotate, and were held fixed relative to the allocentric world frame (see Fig. 4a, right; and Supplementary Fig. S5). The rolled scenario was then frozen, and the subjects performed the exact same task as before—i.e. they had to adjust the ‘vertical’ shift (above the horizon in Fig. 4a-right and above the head in Fig. 4a-left) of the ‘vertical’ target, in order to make it perceptually equal to the ‘horizontal’ shift of the (fixed) ‘horizontal’ target. The roll trials were randomly interleaved with the main trials of Experiment 2 (straight-pose trials) and control trials that were described above.

### Defining the compression ratio

We defined the compression ratio as follows:

1 $$\mathrm{CR}= \frac{(\frac{{\mathrm{\varphi }}_{\mathrm{real}}}{{\mathrm{\varphi }}_{\mathrm{estimated}}})}{(\frac{{\uptheta }_{\mathrm{real}}}{{\uptheta }_{\mathrm{estimated}}})}.$$

In the traveled-space experiment (Experiment 1), φ_real_ = θ_real_ by definition—because in the analysis, we compared the estimated vertical angle to the estimated horizontal angle when the real angles were equal, i.e. when φ_real_ = θ_real_ (e.g. we compared the estimated angles for φ_real_ = θ_real_ = 45°, etc.). Hence, in the traveled space experiment, Eq. (1) reduces to: CR = θ_estimated_/φ_estimated_. In the surrounding-space experiment (Experiment 2), φ_estimated_ = θ_estimated_ by definition—because the subjects were asked to move the vertical angle to be equal to the horizontal angle: i.e. to move it until they estimated that φ_estimated_ = θ_estimated_. Hence, in the surrounding-space experiment, Eq. (1) reduces to: CR = φ_real_ / θ_real_, where φ_real_ is defined as the final vertical angle, φ_final_, at which the subject placed the vertical target (Supplementary Fig. S3, panel 5).

### Statistical tests

We used standard statistical tests, using Matlab. All the test details and their results are reported in the text or in the figure captions.

### Models of egocentric and allocentric perception

The anisotropic perception of space can be described by an ellipse, where the compression ratio, CR, describes the inverse of the eccentricity of the ellipse (see Supplementary Fig. S5). When the subjects were rotated, several scenarios could have described the visual perception: (i) The ellipse rotates with the subject (Supplementary Fig. S5, bottom row, green ellipses); or (ii) The ellipse is fixed to the horizon, i.e. to the allocentric world (Supplementary Fig. S5, bottom row, magenta ellipses); or (iii) Some intermediate situation. Using the geometry of a rotating ellipse, we derived a formula (see below) that predicts the compression ratio under scenarios (i) and (ii) that were described above (targets rotating with subject, and targets fixed relative to the horizon). For the first experimental scenario (Fig. 4a, bottom-left), where targets rotated with the subject, our model provides a prediction for the dependence of the compression ratio on the roll angle, $\mathrm{\alpha }$. If the anisotropy is yoked to the egocentric frame, we expect to find:

2 $$\mathrm{CR}\left(\mathrm{\alpha }\right)=\mathrm{CR}\left(\mathrm{\alpha }=0\right)=\mathrm{constant}.$$

By contrast, for the same experiment, if the anisotropy is yoked to the allocentric frame of reference (the horizon), the compression ratio would be given by:

3 $$\mathrm{CR}\left(\mathrm{\alpha }\right)=\sqrt{\frac{{\mathrm{CR}}^{2}\left(\mathrm{\alpha }=0\right){\mathrm{cos}}^{2}\mathrm{\alpha }+{\mathrm{sin}}^{2}\mathrm{\alpha }}{{\mathrm{CR}}^{2}\left(\mathrm{\alpha }=0\right){\mathrm{sin}}^{2}\mathrm{\alpha }+{\mathrm{cos}}^{2}\mathrm{\alpha }}.}$$

In the complementary experimental scenario (Fig. 4a, bottom-right), where targets did not rotate with the subject but rather were fixed to the horizon, the inverse holds. In this case, Eq. (2) gives the prediction for the allocentric model, while Eq. (3) gives the prediction for the egocentric model. We note that for each subject, the model was fitted with the subject’s own $\mathrm{CR}\left(\mathrm{\alpha }=0\right)$ (Fig. 4b, magenta and green curves; and Fig. 4d)—while for the population average we used the mean value of $\mathrm{CR}\left(\mathrm{\alpha }=0\right)$ (Fig. 4c, magenta and green curves).

## Supplementary Information

Supplementary Information.

## Data Availability

All the data in this study were analyzed and plotted using custom code written in Matlab. The data and code are archived on the Weizmann Institute of Science servers, and will be made available upon a reasonable request from the corresponding author, Nachum Ulanovsky (nachum.ulanovsky@weizmann.ac.il).
