# Supplementary material for: Dissociating two aspects of human 3D spatial perception by studying fighter pilots
Source: Sci Rep. 2023 Jul 12;13:11265. doi: 10.1038/s41598-023-37759-w (PMC10338539; doi:10.1038/s41598-023-37759-w)
Supplement: Supplementary file 1 — Supplementary Information. [file 41598_2023_37759_MOESM1_ESM.pdf]

Supplementary Figures and Tables

Supplementary Figures:

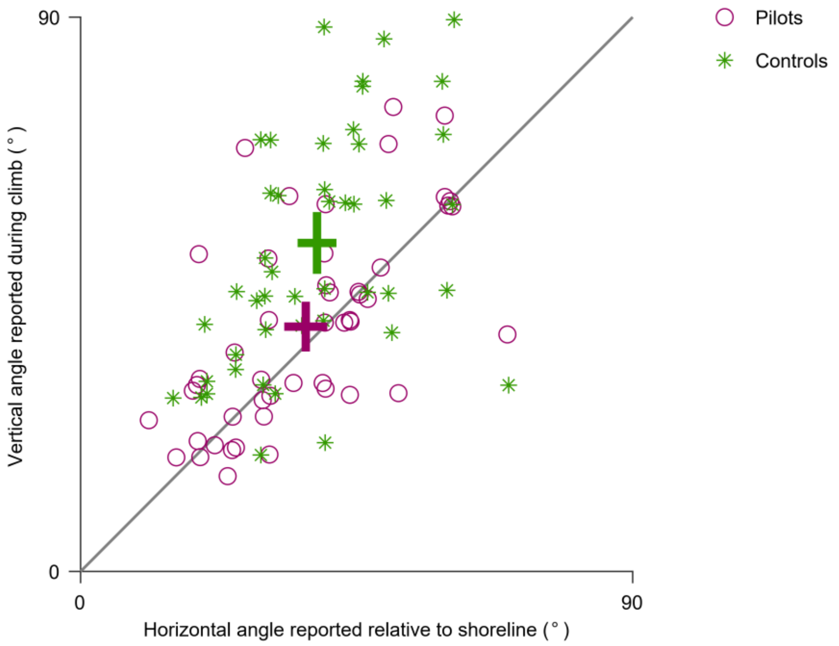

Supplementary Fig. S1. In Experiment 1 (traveled space), pilots estimated vertical and horizontal angles of traveled space in an un-distorted manner, while control subjects overestimated the vertical angle compared to the horizontal angle. Shown is a scatter plot of the vertical versus horizontal angles reported by the subjects, when flown in the same angles in the traveled-space experiment (Experiment 1). Markers depict the data for each subject (three markers per subject), for flown angles of 30°, 45° and 60° (circles – pilots, stars – controls; markers were slightly jittered [Gaussian jitter with standard-deviation of 1°], for display purposes only, to avoid overlaps). The diagonal identity line is depicted in gray. Note that the pilots' markers lie close to the identity line, consistent with isotropic perception of CR = 1; by contrast, the control subjects' markers are largely above the identity line, consistent with anisotropic perception of CR < 1 (see Methods). The two crosses show the mean ± s.e.m. for each population in each of the axes.

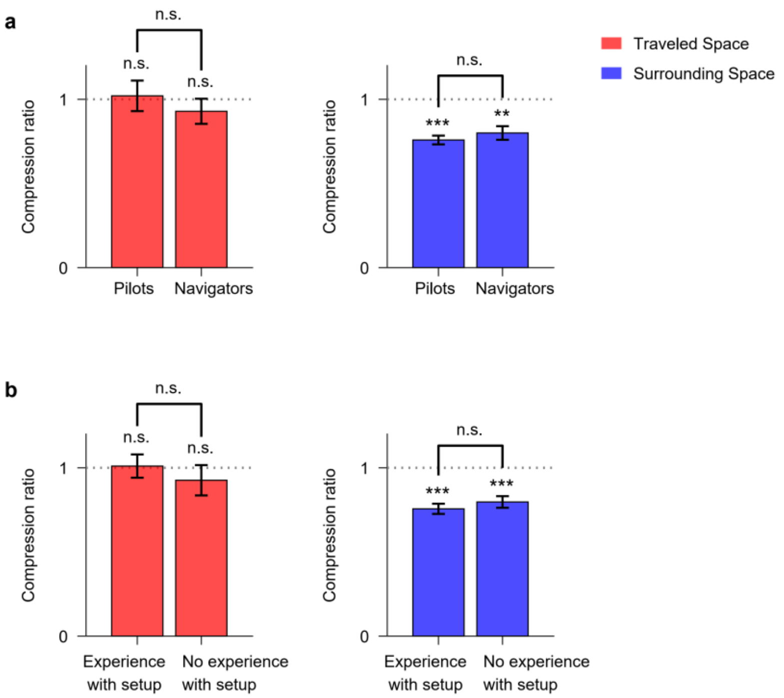

Supplementary Fig. S2. In both types of experiments, we found similar compression ratios (CR) for pilots and navigators within the pilots group, and no effect of prior experience with the setup. a Comparison between the compression effect for the subjects that comprised the 'pilots' test group: the pilots (n = 9) and navigators (n = 7). Comparisons are shown here for traveled-space (left) – the same experiment as in main Fig. 2c, and for surrounding-space (right) – the same experiment as in main Fig. 3c. Results were the same for both pilots and navigators: there was no significant difference between the two subgroups, in either the traveled-space or surrounding-space experiment (comparing each groups' CR to 1: paired t-tests; comparing between groups: unpaired t-tests; '**', $P < 0.01$; '***', $P < 0.001$; 'n.s.', $P > 0.05$) – hence we pooled them together into one 'pilots' group throughout the paper. **b** Comparison between the compression effect for the pilots and navigators that had previous experience with the specific flight simulator setup used in this experiment (n = 8), and those that did not (n = 8). Results indicated that prior experience with the specific flight simulator did not affect the compression ratios: there was no significant difference between the two subgroups, in either the traveled-space or surrounding-space experiment (statistics as in panel a). Error bars, mean ± s.e.m.; dotted gray lines indicate CR = 1.

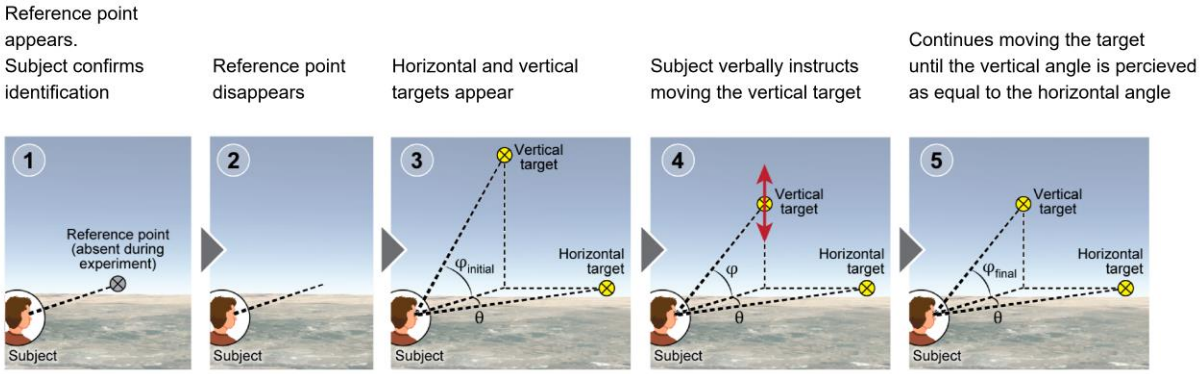

Supplementary Fig. S3. A cartoon depicting the experimental design for testing the perception of 3D surrounding space (Experiment 2). Each experimental trial proceeded in five steps: (1) The subject sat facing the screen, looking at a reference point straight ahead. (2) The reference point disappeared. (3) Two targets appeared: a horizontal target at a horizontal shift θ from the straight-ahead reference point, and a vertical target at a vertical shift φ from the straight-ahead reference point (which had already disappeared at this stage). The subject was asked to look around and identify the targets. (4) During the experiment, the subject was asked to verbally instruct the experimenters to move the vertical target up or down, while the horizontal target remained stationary – this continued until: (5) the subjects reported that they perceived the vertical shift of the vertical target (φ_{final}) to be identical to the horizontal shift of the horizontal target (θ). We quantified vertical versus horizontal perception with a compression ratio (CR), as follows: $CR = \varphi_{final} / \theta$.

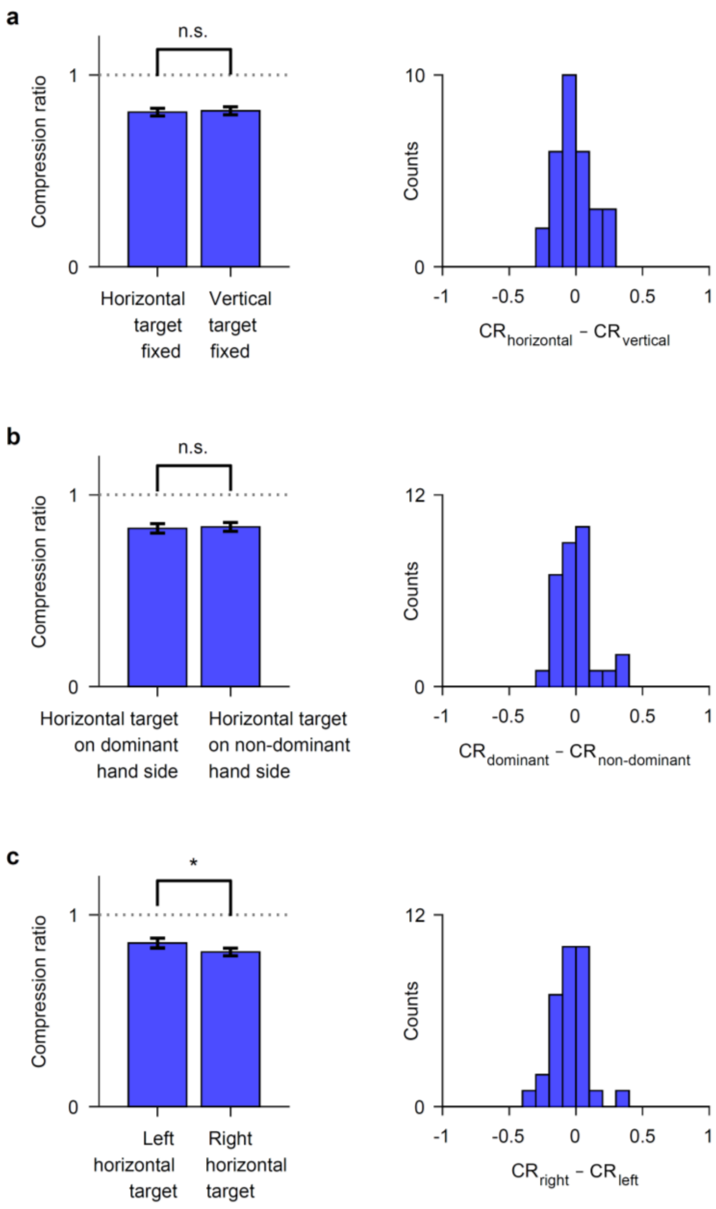

Supplementary Fig. S4. The distorted perception of 3D surrounding space did not depend on whether the movable target was vertical or horizontal, nor on the side of the horizontal target. Controlling for different experimental variables of Experiment 2, we compare here the compression ratios of estimating 3D surrounding space between the main experiment and three different control scenarios. **a** Control 1 – Varying which target (vertical or horizontal) was being adjusted: comparison of the main experiment, in which the horizontal target was fixed and the vertical target was being adjusted, to a control scenario, in which the vertical target was fixed and the horizontal target was being adjusted. Left panel: Bar graph. Compression ratio of

assessing 3D surrounding space was compared between two scenarios: horizontal target was held fixed and the vertical target was adjusted to match it (left bar: this is the standard experiment); or the vertical target was held fixed and the horizontal target was adjusted to match it (right bar: control experiment). Error bars, mean ± s.e.m. (n = 32 subjects; two-sided paired t-test, t = 0.52, P = 0.6; Wilcoxon paired sign rank test, P_{wilc} = 0.39); dotted gray line indicates CR = 1. Right panel: Histogram depicting the difference between the compression ratios for the two scenarios, per subject (same data as on the left, plotted as a histogram of differences). **b** Control 2 – Controlling for the handedness of the subject relative to target: horizontal target was presented on the side of the subject's dominant hand versus on the side of the non-dominant hand. Left panel: Bar graph. Horizontal target was on the dominant side of the subject (left bar) versus on the non-dominant side of the subject (right bar). Error bars, mean ± s.e.m. (n = 32 subjects; two-sided paired t-test, t = –0.32, P = 0.74; Wilcoxon paired sign rank test, P_{wilc} = 0.37); dotted gray line indicates CR = 1. Right panel: Histogram depicting the difference between the compression ratios for the dominant and non-dominant sides, per subject (same data as on the left, plotted as a histogram of differences). **c** Control 3 – Varying the side of the target: comparison of the main experiment (horizontal target was presented on the right side of the subject) to a control scenario in which the horizontal target was presented on the left side. Left panel: Bar graph. Horizontal target was on the left side of the subject (left bar) versus on the right side of the subject (right bar). Error bars, mean ± s.e.m. (n = 32 subjects; two-sided paired t-test, t = –2.03, P = 0.0507; Wilcoxon paired sign rank test, P_{wilc} = 0.023); dotted gray line indicates CR = 1. Right panel: Histogram depicting the difference between the compression ratios for horizontal target located on the left and on the right, per subject (same data as on the left, plotted as a histogram of differences).

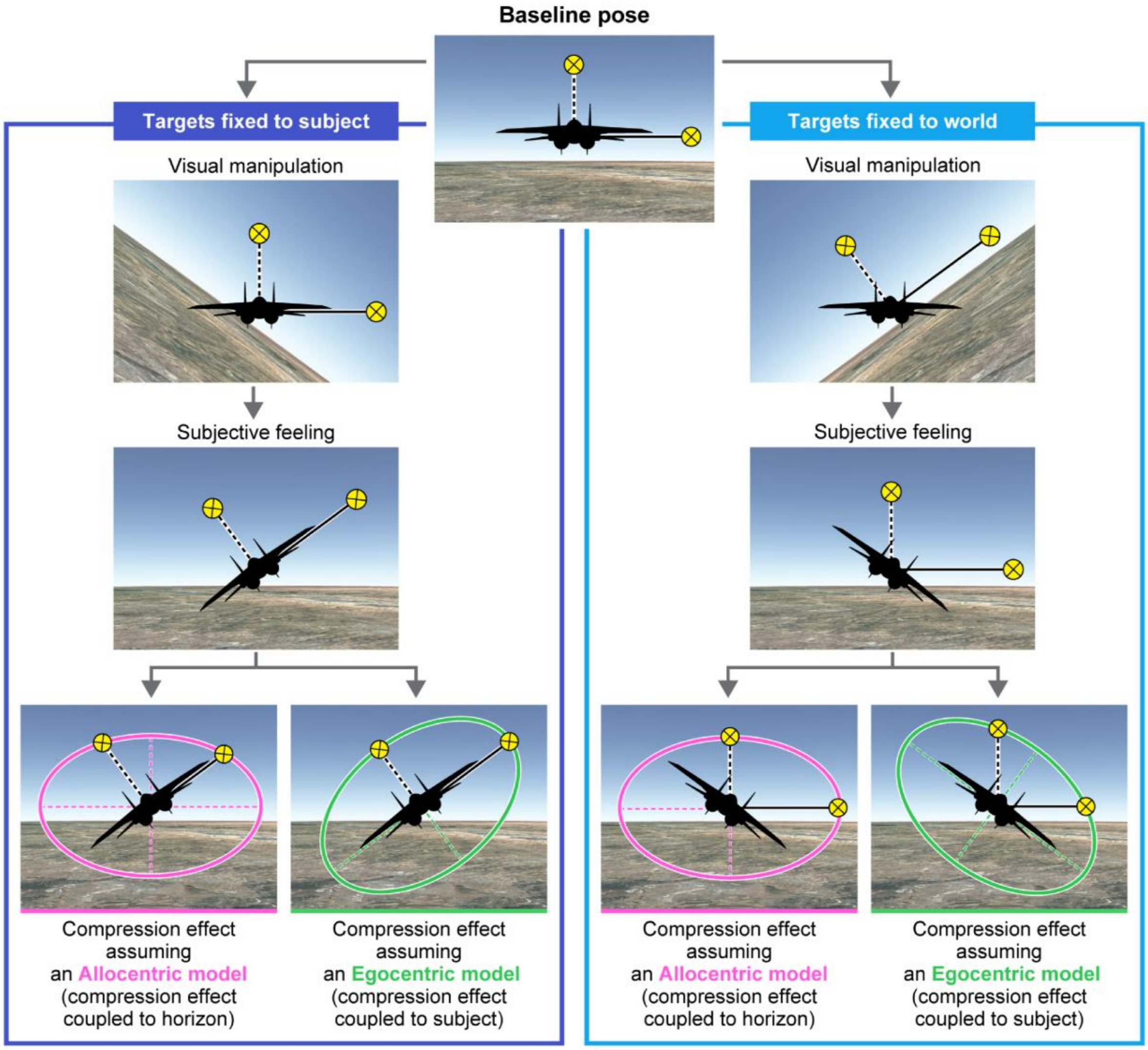

Supplementary Fig. S5. Illustration of the allocentric versus egocentric models. A cartoon describing the experiment that tested egocentric versus allocentric compression effects in the surrounding-space experiment (Experiment 2).

Top middle – the experimental design of the main Experiment 2, as depicted in Fig. 3.

Two columns (dark blue and light blue) – two different roll manipulations that were done to distinguish between egocentric and allocentric effects. *Top of each column*: the immersive visual manipulation done on the subjects sitting inside the stationary cockpit. *Middle of each column*: The subjective feeling of a roll maneuver that the visual manipulation elicited in the subjects. In both manipulations the visual display of the horizon was tilted to create a subjective feeling of doing a

roll maneuver with the airplane: (i) Left column (dark blue): The subjects rolled and the targets rolled with them (so that a target that was above their head before the roll continued to be above their head after the roll). (ii) Right column (light blue): The subjects rolled, but the targets did *not* roll with them, and instead stayed at their original position with respect to the world (so that a target that was above the subjects' head before the roll was *not* above their head after the roll). *Bottom row of each column* – for each roll manipulation, the compression effect was analyzed under two scenarios: when the compression effect is allocentric – tied to the coordinate frame of the world (bottom row, magenta ellipses), and when the compression effect is egocentric – tied to the coordinate frame of the subject (bottom row, green ellipses).

Supplementary Tables:

Trial no. (executed pseudo-randomly)	Testing variable	Vertical angle (°)	Horizontal angle (°)
1	Vertical (Climb)	30	0
2	Vertical (Climb)	45	0
3	Vertical (Climb)	60	0
4	Horizontal	0	30
5	Horizontal	0	45
6	Horizontal	0	60

Supplementary Table S1. Order of trials - Traveled space experiment. A list of the trials conducted in Experiment 1 – Traveled space. Each trial was preceded with a baseline trial of 5–20 second of 'straight and level' horizontal flight along Israel's Mediterranean shoreline, with vertical and horizontal angles both equal to 0°. A randomly-chosen trial was then executed, lasting 0.5–1 minutes per trial. The trials used for this study consisted of either (i) a vertical climb at a fixed angle (vertical angles of 30°, 45° or 60°, trials no. 1–3 in the table); or (ii) a straight-and-level horizontal flight at a particular azimuth relative to the well-defined and recognizable sea shoreline (horizontal angles of 30°, 45° or 60°, trials no. 4–6 in the table). We conducted also additional trials (inverted flights, descent flights [negative climb]) that were not analyzed in this study.

Trial no. (executed pseudo-randomly)	Airplane pose	Targets fixed to	Target to adjust	Roll angle of Airplane relative to ground (°)	Roll angle of Targets relative to airplane (°)	Angle of reference target (°)	Comments
1	Baseline	Subject & world	Vertical	0	0	40	**Main experiment**
2	Baseline	Subject & world	Vertical	0	0	40	
3	Baseline	Subject & world	Vertical	0	0	40	
4	Baseline	Subject & world	Horizontal	0	0	40	**Control experiment** Effect of the adjustable target being vertical/ horizontal
5	Baseline	Subject & world	Vertical	0	0	–40	**Control experiment** Effect of the adjustable target being left/right of the subject, and effect of handedness
6	Roll maneuver	Subject	Vertical	22	0	40	**Egocentric/Allocentric experiment** Test of CR in an egocentric framework
7	Roll maneuver	Subject	Vertical	45	0	40	
8	Roll maneuver	Subject	Vertical	67	0	40	
9	Roll maneuver	Subject	Vertical	90	0	40	
10	Roll maneuver	World	Vertical	11	–11	40	**Egocentric/Allocentric experiment** Test of CR in an allocentric framework
11	Roll maneuver	World	Vertical	22	–22	40	
12	Roll maneuver	World	Vertical	45	–45	40	
13	Roll maneuver	World	Vertical	67	–67	40	
14	Roll maneuver	World	Vertical	90	–90	40	

Supplementary Table S2. Order of trials - Surrounding space experiment. A list of the trials conducted in Experiment 2 – Surrounding space. A total of 14 trials per subject; each trial lasted 2–4 minutes. Trials were pseudo-randomly alternated, interchanging randomly between the main experiment (trials no. 1–3), control trials (trials no. 4–5), and roll trials which tested egocentric versus allocentric reference frames (trials no. 6–14). Participants were seated inside the cockpit, with two targets appearing on the screen surrounding them: a vertical target that was shifted upwards, above eye-level, and a horizontal target that was horizontally shifted to the side of the subject. In each trial, the horizontal target was placed at θ = 40° (or θ = –40° in control

trial no. 5), and the vertical angle $\varphi_{initial}$ was chosen at random with a value that greatly differed from θ (see Methods). Participants were asked to verbally instruct the experimenters by how much to change the vertical target's position (or the horizontal target's position in control trial no. 4), and could continue to fine-tune the vertical target's position until the subjects declared that they perceive the vertical shift as identical to the horizontal shift.
